# Effect of Feed Supplementation with *Clostridium butyricum*, Alone or in Combination with Carob Meal or Citrus Pulp, on Digestive and Metabolic Status of Piglets

**DOI:** 10.3390/ani11102924

**Published:** 2021-10-10

**Authors:** Marina López, Josefa Madrid, Fuensanta Hernández, Martín Antonio Ros, Juan Carlos Segura, Miguel José López, Francisco José Pallarés, Cristian Jesús Sánchez, Silvia Martínez-Miró

**Affiliations:** 1 Department of Animal Production, Faculty of Veterinary Science, Regional Campus of International Excellence “Mare Nostrum”, University of Murcia, 30100 Murcia, Spain; marina.lopez9@um.es (M.L.); nutri@um.es (F.H.); mjlopeza@um.es (M.J.L.); cristianjesus.sanchez@um.es (C.J.S.); silviamm@um.es (S.M.-M.); 2 Agrarian Transformation Society, Number 2439, La Hoya, 30816 Lorca, Spain; mros@alia.es (M.A.R.); jcsegura@alia.es (J.C.S.); 3 Department of Anatomy and Comparative Pathology and Toxicology, Faculty of Veterinary Medicine, Agrifood Campus of International Excellence–ceiA3, University of Córdoba, 14014 Córdoba, Spain; fpallares@uco.es

**Keywords:** piglets, *Clostridium butyricum*, carob meal, citrus pulp, digestive status, metabolic status

## Abstract

**Simple Summary:**

During the intensive production of weaned piglets, frequent digestive disorders need to be avoided, as it is a critical phase; however, there are limitations to using antibiotics and ZnO at high levels. In this study, we investigate the inclusion of a probiotic (*Clostridium butyricum*) in combination with sources of fiber that might have a potential prebiotic effect, generating an optimal digestive status for weaned piglets. A trial is carried out using 30 post-weaning piglets for 27 days using five dietary treatments: a negative control, a positive control with high levels of ZnO, and three dietary treatments supplemented with *Clostridium butyricum* (alone or in combination with carob meal or citrus pulp). Supplementation with this probiotic could improve the piglets’ intestinal wellness status by increasing butyric acid, without being altered by the inclusion of carob meal or citrus pulp at 5%, obtaining digestibility values comparable with those realized by the incorporation of high levels of ZnO in the diet. In addition, carob meal could decrease the concentration of serum interleukin-8 (a type of pro-inflammatory cytokine). However, a growth performance trial of piglets in commercial conditions needs to be developed to confirm these effects.

**Abstract:**

This work studied the effects of the inclusion of *Clostridium butyricum* on feed, alone or with carob meal or citrus pulp, on the digestive and metabolic status of weaned piglets. A total of 30 male piglets (weaned at 21 days) is used. There are five dietary treatments: negative without ZnO at high doses (C−), a positive control supplemented with ZnO at 2500 ppm of Zn (C+), supplemented with *Clostridium butyricum* as a probiotic (PRO), and supplemented with probiotic and 5% carob meal (PROC) or 5% citrus pulp (PROP). During the experiment (27 days), the piglets were periodically weighed and sampled for a serum biochemical, fecal microbiological, intestine histological, and digestive status analysis. The body weight, apparent ileal digestibility of dry matter (DM), and fecal microbiology were not affected by the treatments (*p* ≥ 0.05). However, the apparent fecal digestibility of DM was lower for the C− treatment than for C+ (*p* < 0.05), and the total concentration of volatile fatty acids (VFAs) in feces with C+ was lower than that for the PROC treatment (*p* < 0.05). The treatments with the probiotic had a higher molar proportion of butyric acid in feces than C+, and it was found that C− reached an intermediate value (*p* < 0.01). No general effects of diet were found on the histological measures performed on the jejunum and ileum, and in the serum biochemical analysis (*p* ≥ 0.05), only the concentration of interleukin-8 was lower for the PROC treatment compared to the C−, C+, and PRO treatments (*p* < 0.05). In conclusion, the intestinal wellness of piglets could be improved with the supplementation of *Clostridium butyricum* by increasing butyric acid, and this effect was not altered with the inclusion of carob meal or citrus pulp. More studies under commercial conditions are needed, as the effects might be different in more challenging environmental circumstances.

## 1. Introduction

The weaning of piglets is a critical period due to the dietary challenges for the pigs, and the social and environmental changes to which they are subjected [1]. Antimicrobial compounds have been widely used in feed for weaned piglets to improve performance and to prevent diarrhea [2]. However, concerns regarding the use of antibiotics have grown, due to the possible consequences that antibiotic resistance can have on human and animal health [3]. The European Union (EU) implemented a ban on using antibiotics as growth-promoting additives in non-medicated feeds for livestock from January 2006; they currently have a limited use even in medicated feeds. In addition, other products used to prevent digestive problems in piglets, based on zinc or copper, have been restricted due to the environmental problems that might be resulted [4]. Furthermore, feed supplementation with high levels of zinc oxide (ZnO) has been related to the development of antibiotic-resistant microorganisms in piglets [5]. However, ZnO has been widely used at high levels (2500 ppm of Zn) as a strategy to prevent diarrhea in piglets, but the European legislation has set a target of zero ZnO usage in pharmacological doses in piglet feed by 2022 [6]. In this context, searching for alternative strategies to modulate the intestinal microorganisms of weaned piglets is a research goal. Among these alternatives are probiotics [7], which are harmless live microorganisms that balance intestinal microbiota for the benefit of the animal [8]. An example is the preparation *Clostridium butyricum* FERM-BP 2789, which was authorized as a zootechnical feed additive for weaned piglets (Regulation (EU) No 373/2011) [9]. This bacterium can produce butyric acid, which is an energetic substrate for intestinal epithelium cells and a regulator of intestinal pH; consequently, it can preserve an optimal intestinal environment [10]. In piglets, some studies have shown that diet supplementation with *Clostridium butyricum* can improve the immune status, the structure of the intestinal mucosa, and the microbial profile of the intestine [11], resulting in an improvement of the feed conversion ratio [12]. This additive has also had positive effects during the weaning of piglets in combination with other probiotics [13,14]. However, there is little information on the use of *Clostridium butyricum* in combination with other substances with an additive or synergistic action that could improve the potential results of its use. In this way, the combination of probiotics with prebiotics is a strategy that is currently being studied [15]. A prebiotic is a food ingredient, totally or partially non-digestible, which can ferment in the digestive tract increasing the growth of the bacteria that could improve the health of the host [16]. Some fiber sources have shown prebiotic effects [17]. In post-weaning piglets, ingredients such as wheat bran and pea fibers have been able to modulate the intestinal microorganisms by stimulating the beneficial bacterial species, although others such as maize fiber and soyabean fiber did not show any positive effects [18].

Carob meal (originally from the fruit of *Ceratonia siliqua*) and citrus pulp are two fiber-rich by-products typical of the Mediterranean area having different fiber profile compositions [19], with carob meal having a higher neutral detergent fiber (NDF) and lignin content. In addition, carob pods are sweet with high levels of sucrose [20], giving it a more palatable characteristic, and carob is rich in tannins that confer astringent properties (preventing diarrhea), being a natural source of antioxidants [21]. Tannins (polyphenolic compounds), present in carob and its derivatives, are considered powerful antioxidants and anti-inflammatories, having bacteriostatic and antidiarrheal properties that could be of interest for piglet diets [22]. In addition, Andrés-Elias et al. [23] observed that the incorporation of carob in diets for piglets could affect the intestinal microbiota. Lizardo et al. [24] studied the incorporation of carob meal in the feed of weaned piglets and found that it did not affect the productive parameters, and the prevalence of post-weaning diarrhea dropped by 20–33% when carob meal was included at 3% and 6%, respectively.

Citrus by-products are rich in pectins (fermentable fiber) and, in addition, they have available biologically active compounds, such as polyphenols, carotenoids, and essential oils [25]. Furthermore, Hotchkiss et al. [26] concluded that pectic oligosaccharides from citrus fruits had prebiotic bifidogenic properties. In piglets, Pascoal et al. [27] indicated that a 9% citrus pulp inclusion decreased the occurrence of *E. coli* in the small intestines of piglets. Moreover, Collier et al. [28] showed that in newly weaned pigs challenged by *E. coli* F18, a 10% citrus pulp inclusion suppressed the ileal and cecal recovery of this pathogen. However, some studies on citrus pulp incorporation showed negative effects on nutrient utilization (at 4.5% of inclusion) [29], and on incidences of diarrhea [30] or negative effects on performance [27] (at 9% of inclusion); although, others authors did not find negative effects on performance when they included 7.5% citrus pulp in the diets of weaned piglets [31]. Therefore, there is some controversy regarding the use of this ingredient in piglets.

Thus, beyond the traditional concept of using a prebiotic to enhance the action of a specific probiotic, the hypothesis of the present research is that the inclusion of a probiotic such as *Clostridium butyricum*, in combination with sources of fiber that could have a positive complementary effect, could generate a healthy digestive environment and improving the digestibility and physiological status of weaned piglets, but this effect could be different depending on the type of fiber ingredient. Therefore, the objective of the present work is to evaluate the effect of the inclusion of *Clostridium butyricum*, alone or with carob meal or citrus pulp, on the digestive and metabolic status of post-weaning piglets in optimal nursery conditions.

## 2. Materials and Methods

All experimental procedures performed in this work were in compliance with the protection of animals used for scientific purposes regulated by the European Union (2010/63/EU Directive) [32]. The administrative authorities and the Ethics Committee of the University of Murcia (Murcia, Spain) approved the protocol (code A13170502).

### 2.1. Animal and Experimental Design

The study was conducted for 27 days in the Animal Nutrition Experimental Unit at the veterinary farm of the University of Murcia (Guadalupe, Murcia, Spain) in optimal nursery conditions. A total of 30 piglets (non-castrated male, 100% Large White breed) were weaned at 21 days old, individually identified, and weighed. The piglets had an average body weight (BW) of 5.06 ± 0.64 kg. They were housed in a controlled environment nursery, and were randomly allotted to one of the five dietary treatments, with six piglets per treatment. Each dietary treatment group was housed in a pen with a plastic slat floor and a space of 0.5 m^2^ per animal, equipped with feeders and nipple drinkers, offering ad libitum access to feed and water throughout the experiment.

The diets consisted of two control treatments, one negative without ZnO at high doses (C−), and one positive control (C+) consisting of a basal diet with ZnO added at a high level (2500 ppm of Zn), and another three experimental treatments, one only supplemented with *Clostridium butyricum* (*Clostridium butyricum Miyairi* 588 (FERM BP-2789), MIYA-GOLD^®^ S, Huvepharma^®^, Antwerp, Belgium) to provide 2.5 × 10^8^ CFU per kg feed (PRO), and another two with the same probiotic plus 5% carob meal (PROC) or 5% citrus pulp (PROP). The commercial additive with *Clostridium butyricum* was included at 0.05%, during the mixing of ingredients, to provide the feeds with this probiotic, at the established dose. The carob meal or citrus pulp were incorporated by substituting all of the sugar beet pulp and part of the barley of a basal diet, and carefully balancing the other minor ingredients until all diets were iso-energetic, iso-aminoacidic, and iso-neutral detergent fiber (iso-NDF). All experimental feeds were formulated to meet or exceed the requirements of piglets as indicated by the Spanish Foundation for the Development of Animal Nutrition (Fundación Española para el Desarrollo de la Nutrición Animal, FEDNA) [33]. In addition, 0.5% TiO_2_ was included in all diets as an indigestible marker to determine the apparent fecal and ileal digestibility of the feeds (Table 1). The feeds were manufactured by Agrarian Transformation Society number 2439 (La Hoya, Spain) from the same ingredient batches. The diets were presented in mash form. In addition, the body weight (BW) of the piglets was controlled during the experiment on days 0, 14, and 26.

### 2.2. Sampling Collection

On day 25 of the experiment, after a period of 8 h of fasting, blood samples were obtained from the jugular vein of the piglets by venipuncture, using a 4 mL vacuum tube per animal (Z Serum Clot Activator, VACUETTE^®^, Greiner Bio-One GmbH, Kremsmünster, Austria). Next, in the laboratory, they were centrifuged at 2500× *g* for 10 min to obtain serum, which was stored in aliquots at −80 °C for the subsequent determination of the blood biochemical profile and cytokines. In addition, individual fecal samples were collected on day 26 of the trial directly from the anus of each piglet and placed in a sterile bottle for fecal microbiology analysis.

On the last day of the trial (day 27), prior to animal slaughtering, individual fecal samples were collected for chemical analysis, then the piglets were tranquilized with an intramuscular injection of azaperone, and later euthanized with an intravenous overdose of pentobarbital. They were bled, and their abdomens were immediately opened by an incision from sternum to pubis to remove the entire digestive tract. Samples were taken from the content of the stomach, duodenum, jejunum, ileum, cecum, and colon. The pH of these samples was immediately determined from the different sections of the gastrointestinal tract by insertion of a pH meter electrode (pH-Meter GLP 21, Crison Instruments, S.A., Alella, Barcelona, Spain), except for the colon where the pH determination was recorded according to the procedure described by Peters et al. [34]. Additionally, the ileal content was collected for chemical analysis.

The fecal and ileal content were lyophilized and stored at −20 °C in airtight containers until TiO_2_ analysis in the laboratory. In addition, sub-samples of fecal and cecum content (1 g) were acidified with 0.032 mL of H_2_SO_4_:H_2_O (50:50) dilution and stored at –20 °C until volatile fatty acid (VFA) analysis.

For histomorphometrical and immunohistochemical analysis, samples of the piglets’ middle section of jejunum and ileum were obtained (2 cm) and fixed in 10% buffered formaldehyde and, subsequently, embedded in paraffin wax.

### 2.3. Analysis of Feed, Digesta, and Feces

The feed samples were ground to pass through a 1 mm sieve in a laboratory mill (RETSCH ZM 200 Ultra Centrifugal Mill; RETSCH, Hann, Germany). These samples were analyzed using the Association of Official Analytical Chemists (AOAC) procedures [35]: dry matter (DM) by the 934.01 method; crude protein (CP) by the 2001.11 method. Van Soest et al. [36] procedures were used to determine the neutral detergent fiber (NDF) and acid detergent fiber (ADF), analyzing the acid detergent lignin (ADL) through the solubilization of cellulose with 72% H_2_SOH_4_.

Sub-samples of the feeds were ground to pass a 0.5 mm sieve (in the same mill indicated above) to analyze the total, insoluble, and soluble dietary fiber using the AOAC 991.43 enzymatic–gravimetric method with the Megazyme K-TDFR-100A/K-TDFR-200A 04/17 kit (Megazyme Ltd., County Wicklow, Ireland).

Furthermore, the marker (TiO_2_) used to calculate digestibility was analyzed in the feeds, feces, and ileum contents using the method described by Myers et al. [37]. For this analysis, the samples were also ground to pass through a 0.5 mm sieve. Feces and ileal contents were also analyzed for CP, as it was indicated previously.

The VFA concentration in the cecum and feces was determined by capillary gas chromatography using an adaptation of the method described by Madrid et al. [38]. The gas chromatograph equipment used was a TRACE GC Ultra (Thermo Finnigan Italia SpA, Milan, Italy) with a flame ionization detector, and the capillary column was fused silica (30 m × 0.25 mm × 0.25 μm ID) coated with FFAP-TR as the stationary phase (Teknokroma, Barcelona, Spain). Standard solutions of acetic, propionic, butyric, isobutyric, isovaleric, and valeric acids were prepared for calibration, using 3-methyl-n-valeric acid as the internal standard as indicated by Oliveira et al. [39].

One gram of each collected fecal sample for microbial study was diluted with a sterile saline solution in a 1:10 dilution and homogenized on a mixer-homogenizer for 2 min. Subsequently, aliquots of the ten-fold serial dilutions were spread-plated onto selective media. The dilutions were used for counting *Enterobacteriaceae*, coliforms, and lactic acid bacteria. Thus, the *Enterobacteriaceae* counts were determined by the ISO 21528-2:2004 [40] adapted method (RAPID’ Enterobacteriaceae/Agar, Bio-Rad Laboratories, S.A., Madrid, Spain) (at 37 °C for 24 h). Coliform bacteria were enumerated using violet red bile lactose (VRBL) agar (at 37 °C for 24 h) according to ISO 4832:2006 [41]. Mesophilic lactic acid bacteria were enumerated using De Man, Rogosa, and Sharpe (MRS) agar (at 30 °C for 72 h) following ISO 15214:1998 [42] and the incubation was carried out with a double-layer MRS medium to provide anaerobic conditions. The results were expressed as log_10_ CFU (colony-forming units)/g of feces.

### 2.4. Histomorphometrical and Immunohistochemical Procedures

Sections (4 µm) of each intestinal tissue sample were stained with hematoxylin and eosin for the morphometric study. For this determination, a ZEISS Axioskop 40 microscope (Carl Zeiss, Oberkochen, Germany) was used with a Spot Insight camera, and the Spot Advanced software (Spot Imaging Solution, MI, USA). In each slide, the height and crypt depth of 10 villi were measured and the results were expressed in μm. In addition, the villus height/crypt depth ratio was determined. The number of intraepithelial lymphocytes and goblet cells was quantified by counting in 10 fields of epithelium of 25,000 μm^2^.

For immunohistochemical analyses, the detection of IgA-secretory cells in the jejunum and ileum was performed by the avidin–biotin–peroxidase complex technique, according to Oliveira et al. [39]. In the intestinal lamina propria, the number of IgA-positive cells was checked with a ZEISS Axioskop 40 microscope (Carl Zeiss, Oberkochen, Germany) using a Spot Insight camera and the Spot Advanced software (Spot Imaging Solution, MI, USA). Immunolabeled cells were recorded in 10 non-overlapping consecutive fields of 25,000 μm^2^.

The same researcher, blinded to the treatments, performed the morphometric and immunohistochemical determinations.

### 2.5. Serum Analysis

The serum metabolic profile was analyzed for glucose, urea, total cholesterol, triglycerides, total bilirubin, total protein, albumin, aspartate aminotransferase (AST), and alanine aminotransferase (ALT) using commercially available kits (Beckman Coulter Inc., Fullerton, CA, USA). The total antioxidant capacity (TAC) and total oxidative status (TOS) were measured according to Erel [43,44], using colorimetric assays. All analyses were executed in an automated chemistry analyzer (Olympus AU600, Olympus Europe GmbH, Hamburg, Germany).

Serum cytokine levels were assayed after centrifugation of the samples at 20,000× *g* to eliminate the lipid phase. Interleukin (IL)-1β, IL-6, IL-8, IL-10, IL-12, and tumor necrosis factor-α (TNF-α) were determined using a porcine-specific multiplex cytokine/chemokine assay (cat. no. PCYTMAG-23K; Millipore MILLIPLEX, Billerica, MA, USA) on a MAGPIX instrument (Luminex; Luminex Technologies, Austin, TX, USA), as indicated by the manufacturer.

### 2.6. Calculations and Statistical Analyses

The apparent fecal and ileal digestibility of DM and CP were determined by the digestibility equation as follows:

(1) $$Digestibility\;\left(\%\right)=\left[1-\left(\frac{\%\;TiO_{2}\;in\;feed}{\%\;TiO_{2}\;in\;feces\;or\;ileum\;digesta}\times \frac{\%\;Nutrient\;in\;feces\;or\;ileum\;digesta}{\%\;Nutrient\;in\;feed}\right)\right]\times 100$$

All statistical analyses were performed using the SPSS Statistics software (IBM Corporation, Armonk, NY, USA), with the piglet set as the experimental unit. Data were analyzed by one-way ANOVA. The Shapiro–Wilks test was performed to assess the normality of data from the serum study; a logarithmic scale transformation was performed when the data did not follow normal distribution. Pairwise comparisons of means were performed using the Tukey test. Significance was determined at *p* < 0.05, and a trend was assumed at 0.05 ≤ *p* < 0.10.

## 3. Results

### 3.1. Body Weight, Digestibility, and Digesta pH

The body weights and average daily gains of the piglets were not affected (*p* ≥ 0.05) by the dietary treatments (Table 2). Furthermore, no effect of the feeds on the apparent ileal digestibility of DM was found (*p* ≥ 0.05), although a significant effect (*p* < 0.05) of dietary treatments on the apparent fecal digestibility of DM was observed. Thus, the fecal digestibility of DM was lower for the C− treatment than for C+ (78.93% versus 83.80%, respectively); PRO, PROC, and PROP treatments reached intermediate values, having no differences with C− or C+. In addition, dietary treatments did not affect apparent fecal and ileal digestibility of CP (*p* ≥ 0.05).

The digesta pH of the stomach, duodenum, jejunum, ileum, and colon was not affected (*p* ≥ 0.05) by the treatments. Only a slight trend was detected in the pH of the cecum content (*p* < 0.1), with C+ obtaining the higher value.

### 3.2. Microbiology and VFAs

The *Enterobacteriaceae*, coliform, and lactic acid bacteria counts from the piglet feces were not significantly affected by the dietary treatments (*p* ≥ 0.05) (Table 3).

The total concentration of VFAs in the cecum tended to be different among treatments (*p* < 0.1) (Table 4). The C+ treatment had the lowest level (55.54 mmol/kg), although this value did not differ from the other treatments, except for the C− group, which had the highest cecum VFA concentration (93.44 mmol/kg). In addition, a dietary effect was not observed (*p* ≥ 0.05) on the fermentation profile (VFA molar %) in the cecum.

However, the total VFA concentration in the feces was significantly different among treatments (*p* < 0.05). The VFA concentration in the C+ treatment (66.76 mmol/kg) was at a lower value than in PROC (121.42 mmol/kg), while C−, PRO, and PROP reached intermediate levels. Regarding the VFA molar proportion of the feces, a significant dietary effect on acetic acid was observed (*p* < 0.05). The C+ treatment obtained a higher percentage of acetic acid (74.41 molar %) than PROC (64.26 molar %), with C−, PRO, and PROP having intermediate values. Additionally, significant effects were found on the molar percentage of butyric acid (*p* < 0.01); in this case, the C+ treatment had a lower molar proportion of butyric acid (3.16 molar %) than the three treatments with probiotic (8.67, 8.95, and 7.96 molar % for PRO, PROC, and PROP, respectively), while the C− treatment had an intermediate molar proportion of butyric acid (5.36 molar %), and no differences with the rest of the treatments.

### 3.3. Histomorphometrical and Immunohistochemical Intestinal Measurements

Table 5 presents the effects of the dietary treatments on the histomorphometrical and immunohistochemical intestinal parameters of the piglets. Villi height, crypt depths, and villus height/crypt depth ratios, both in the jejunum and in the ileum of the piglets, were not affected (*p* ≥ 0.05) by the treatments. Similarly, the number of goblet cells and lymphocytes from both intestinal sections were also not affected by the dietary treatments (*p* ≥ 0.05). Furthermore, as a result of the immunohistochemical study of the jejunum and ileum, it was found that the experimental diets had no effect on the IgA cell count (*p* ≥ 0.05).

### 3.4. Serum Parameters

The effects of feeding with experimental diets on the general biochemical parameters, oxidative status indicators, and cytokines in the serum of piglets at day 25 of the trial are displayed in Table 6. In general, no differences were found (*p* ≥ 0.05) in the serum biochemical parameters; only a tendency was observed (*p* < 0.1) in the ALT serum, which reached a higher enzymatic activity in C+ than in the PROC treatment (115.42 versus 71.30 IU/L, respectively), and no differences with the rest of the treatments. In addition, the oxidative status indicators (TAC and TOS) in the serum were not affected (*p* ≥ 0.05) by the dietary treatments.

Regarding the effect of the diets on the serum cytokine level, no significant effects (*p* ≥ 0.05) on IL-1β, IL-6, IL-10, IL-12, or TNF-α were found; however, the IL-8 concentration was significantly (*p* < 0.05) lower for the PROC treatment compared to the C−, C+, and PRO treatments (1.145 versus 3.346, 2.992, and 3.043 ng/mL, respectively), while the PROP treatment obtained intermediate levels (2.219 ng/mL).

## 4. Discussion

Restrictions on high-level ZnO usage in piglet feed is a major challenge for the sustainability of the current levels of intensive pig production [4]. There are many changes related to the handling, environmental, and feeding conditions that need to be addressed. Therefore, with a view to optimizing piglet feed that is free from restricted substances, exhaustive studies need to be performed.

In our experiment, the inclusion of *Clostridium butyricum* in feeds without high levels of ZnO, alone or in combination with carob meal or citrus pulp, did not affect the body weight or weigh gain of piglets after weaning, compared to both control feeds (with or without ZnO). Chen et al. [11] found similar results when weaned piglets were fed *Clostridium butyricum* in increasing doses, compared to a negative control diet. Regarding the inclusion of carob meal, authors such as Špoljarić et al. [45] observed an improvement in the body weight of piglets at 42 days post-weaning when the feed was supplemented with 4% carob whole meal, although this effect was not found at 28 days post-weaning by these authors. The effect of including citrus pulp in feed on the body weight or weight gain of weaned piglets showed varying results, depending on the percentage of the ingredient that was included. Almeida et al. [31] found no negative effect on body weight when they incorporated 7.5% citrus pulp in the feed of weaned piglets, although Pascoal et al. [27] showed negative results on weight gain when this ingredient was incorporated at 9%. It should be highlighted that despite of the fact that our trial diets did not show negative effects on the growth of the animals, they could affect the intake or the feed conversion ratio, so performance tests should be carried out to evaluate these potential effects.

The apparent ileal digestibility (of DM or CP) was not affected by the treatments, although the apparent fecal digestibility of DM improved by 5% with the C+ (high level of ZnO) treatment when compared to C−; in addition, in spite of fecal CP digestibility not being significantly altered by treatments, C+ reached a quantitatively higher value than C−. Dębski [46] indicated that dietary Zn supplementation is used to reduce the fermentation of nutrients in the intestine, improving nutrient digestibility. The inclusion of *Clostridium butyricum*, alone or in combination with carob meal or citrus pulp, did not result in a difference in C+, and neither in C−, where fecal DM digestibility reached intermediate levels. Han et al. [47] suggested that supplementation with *Clostridium butyricum* increased total tract digestibility, as it increased the concentrations of the VFAs that reduce gut pH, achieving an anti-bacterial effect and increasing butyrate, which provides energy for intestinal epithelial cells. In addition, these authors indicated that this effect was more marked when the diet was supplemented with a higher level of probiotic (2.5 × 10^9^ CFU/kg of *Clostridium butyricum*).

We hypothesized that the inclusion of a combination of *Clostridium butyricum* plus carob meal or citrus pulp could generate a positive complementary effect on the intestinal environment. The probiotic could help to improve digestive tract health; and the fiber ingredients could provide an additional favorable environment for beneficial bacteria. In this way, it can be said that, depending on the physicochemical properties of the fiber diet, the effects could be different [48]. Soluble fiber is more fermentable, and it may produce an increase in VFAs that affects the intestinal environment; insoluble fiber may reduce the digesta transit time, preventing the proliferation of pathogenic bacteria [49]. In addition, different effects resulting from the amount, type of fiber, and post-weaning period that were applied have been noted [50]. In our trial, different fiber ingredients were used, but all diets were iso-NDF formulated, resulting in few analytical differences in the feeds, both in the Van Soest fractions and in dietary fiber. No additional improvements in digestibility nor harmful effects were found with the carob meal or citrus pulp at 5% inclusion. On the other hand, when Zhang et al. [51] evaluated the effects of *Clostridium butyricum* and corn bran at 5% on weaned piglets, they observed a decrease in digestibility, and no positive interaction between the corn bran and *Clostridium butyricum*. However, Chen et al. [50] evaluated the effects of dietary soluble and insoluble fiber inclusion (alone or in different combinations) on weaning piglets, and found that all treatments supplemented with fiber presented a higher apparent fecal digestibility of DM than the control group; and the effects on the ileum *Lactobacillus* content and cecum digesta VFAs depended on the type of dietary treatment.

In our study, despite finding that the treatments had no effect on the general population of *Enterobacteriaceae*, coliforms, or lactic acid bacteria in feces, the concentrations of VFAs were affected by the treatments. Therefore, a more exhaustive study of the microbiota is desirable for improving knowledge in this area. We found that the concentration of VFAs in feces with C+ was lower than that in the PROC treatment. O’Shea et al. [52] also found that ZnO in piglet feed decreased the VFA content in feces. They justified this fact with the implication that ZnO could have decreased the secretion of chloride from the colon mucosa; therefore, reducing the secretion of fluids, and contributing to a reduction in water in the digesta and an alteration of the microbial activity. On the other hand, although changes in the VFA profile in the cecum content were not observed, they were found in the feces. Thus, the diets that included the probiotic showed a higher molar percentage of butyric acid, in relation to the diet with high levels of ZnO. *Clostridium butyricum* is a Gram-positive anaerobe that produces butyric acid, which could provide nutrients for the regeneration of intestinal epithelial cells, contributing to intestinal health [53].

It is known that the inclusion of ZnO at high levels increases the height of villi and decreases crypt depth, improving the villus height/crypt depth ratio [54,55], which could be related to a greater capacity for nutrient absorption in the small intestine. However, in our case, any dietary treatment, including the ZnO, did not affect the histomorphology of the intestinal mucosa. Moreover, the number of goblet cells, lymphocytes, and IgA cells in the intestinal mucosa were also not affected. This could be related to the fact that the piglets were not subjected to challenging aggressions that could affect the intestinal mucosa, and they did not manifest diarrhea problems regardless. In this way, Liu et al. [56] indicated that the effect on the intestinal morphology of adding Zn into piglet diets, even at high doses, is limited under optimal physiological conditions.

In general, the dietary treatments in our trial did not affect the serum biochemistry profile of the piglets. In addition, the levels of glucose, urea, cholesterol, triglycerides, total bilirubin, total proteins, albumin, and the albumin/globulin ratio were within the ranges indicated by Perri et al. [57] and Ventrella et al. [58] for piglets close to weaning. The mean levels of ALT and AST enzymatic activity were within the reference ranges indicated by Caprarulo et al. [59], although these were close to the maximum levels observed by Klem et al. [60] for growing pigs. It should be noted that the ALT, a liver enzyme that, at high levels, is considered to be a biomarker of liver damage [61], was different for the PROC and C+ treatments. Neither the TOS or TAC values were affected by the treatment, which was evidenced by the absence of dietary effects on the oxidative status of the piglets. Despite the PROC treatment containing carob, an ingredient with high levels of phenolic compounds, potentially with an antioxidant effect, most of those are in the form of condensed tannins [62], a chemical form which is poorly bioavailable [63,64].

Many studies have determined that, during the inflammation of the gastrointestinal tract, there is an imbalance of the inflammatory cytokine profile [65]. In our experiment, the serum cytokines values were not altered by dietary treatments, except for the concentration of IL-8 in the PROC diet. Zinc at high levels in feeding piglets has shown different results on cytokines. Zhu et al. [55] found differences in the gene expression of certain cytokines from the jejunum mucosa when comparing piglets supplemented with ZnO (3000 ppm) with piglets that were fed a basal diet; specifically, a downregulated IL-1β expression (a pro-inflammatory cytokine) and an upregulated TGF-β expression (an anti-inflammatory cytokine). Similarly, other authors observed a decrease in the expression of other pro-inflammatory cytokines, such as TNF-α, IL-6, and IFN-γ, at day 7 post-weaning in piglets supplemented with high levels of ZnO; however, no differences at day 14 post-weaning were found [66]. In addition, Kloubert et al. [67] studied Zn supplementation at 0, 100, and 2500 ppm in the form of ZnO in weaned piglets, finding that the concentrations of IL-1β, IL-6, or TNF-α in diluted whole blood cultures (incubated with or without substance stimulates of cytokine production such as lipopolysaccharide or phytohemagglutinin) from piglets with dietary treatments were not affected by Zn supplementation. However, IL-2 concentration (a pro-inflammatory cytokine) increased in peripheral blood mononuclear cell cultures (incubated with substance stimulates of cytokine production) from piglets supplemented with 2500 ppm of Zn [67].

Regarding feed supplementation with probiotics, one study showed a decrease in the concentration of IL-1β in the serum of weaned piglets that were supplemented with *Clostridium butyricum* and challenged with enterotoxigenic *Escherichia coli* K88 [68]. In addition, other authors have found changes in the cytokine profile when probiotics were used in the feed of piglets challenged with *Escherichia coli* lipopolysaccharide [69], or affected by post-weaning colibacillosis [70]. Nevertheless, in our study, the general biochemical serum profile and other parameters that were evaluated indicated the absence of nutritional imbalances or harmful processes on the animals’ health. In this sense, as cytokines are produced by the action of a stimulus, it could be hypothesized that, in favorable environmental situations, changes in their concentrations could be difficult to detect.

However, the PROC dietary treatment had the lowest IL-8 cytokine value, which is a type of pro-inflammatory cytokine [65], being lower than that in the C−, C+, and PRO treatments. These results could be related to the inclusion of carob meal as an ingredient, since this feedstuff, or its derivates, has shown anti-inflammatory activity in in vitro assays and animal models [71,72]. Moreover, the advantageous effects of carob products on the other immunity parameters have been suggested. Špoljarić et al. [45] observed that supplementation with carob whole meal did not affect the amount of red blood cells or leucocytes in weaned pigs, but an increase in the proportions of various types of lymphoid cells in the peripheral blood was observed.

## 5. Conclusions

The results of this work showed that supplementation with *Clostridium butyricum* may improve the intestinal wellness status of piglets by increasing butyric acid, without alteration from the inclusion of carob meal or citrus pulp at 5%, obtaining digestibility values comparable with the incorporation of high levels of ZnO in the diet. In addition, carob meal could decrease the concentration of serum IL-8 cytokine. Moreover, further studies on the gut microbiota and growth performance of piglets in commercial conditions would be desirable, as these effects could be different in more challenging environmental conditions.

## Figures and Tables

**Table 1:** Ingredients and composition of experimental diets (as-fed basis).

Item	Diets ^1^				
	C−	C+	PRO	PROC	PROP
Ingredients, %					
Corn flakes	16.00	16.00	16.00	16.00	16.00
Wheat flakes	16.00	16.00	16.00	16.00	16.00
Dehulled barley flakes	8.00	8.00	8.00	8.00	8.00
Corn	9.08	9.08	9.08	9.57	8.77
Wheat	9.90	9.90	9.90	8.00	8.00
Barley	5.00	5.00	5.00	1.50	3.21
Soy-protein concentrate ^2^	7.90	7.90	7.90	7.90	7.90
Soybean meal, 46% crude protein	6.30	6.30	6.30	6.30	6.30
Porcine hydrolyzed protein ^3^	2.19	2.19	2.19	2.87	2.64
Protein concentrate ^4^	2.00	2.00	2.00	2.00	2.00
Sweet whey (dried)	5.00	5.00	5.00	5.00	5.00
Whey powder, 50% fat	1.00	1.00	1.00	2.02	1.26
Dextrose monohydrate	1.40	1.40	1.40	1.40	1.40
Titanium dioxide	0.50	0.50	0.50	0.50	0.50
Soybean oil	2.89	2.89	2.89	3.00	3.10
Sugar-beet pulp	2.00	2.00	2.00		
Carob meal				5.00	
Citrus pulp					5.00
Probiotic ^5^			0.05	0.05	0.05
Zinc oxide ^6^		0.31			
Vitamin–mineral premix ^7^	0.40	0.40	0.40	0.40	0.40
Calcium carbonate	1.00	1.00	1.00	1.00	1.00
Monocalcium phosphate	0.80	0.80	0.80	0.88	0.87
Sodium chloride	0.35	0.35	0.35	0.35	0.35
L-Lysine 50 (50%)	0.96	0.96	0.96	0.96	0.96
DL-Methionine (99%)	0.28	0.28	0.28	0.28	0.28
L-Threonine (98%)	0.31	0.31	0.31	0.31	0.31
L-Tryptophan (98%)	0.10	0.10	0.10	0.11	0.11
L-Valine	0.19	0.19	0.19	0.19	0.20
Sepiolite	0.33	0.02	0.28	0.3	0.27
Mycotoxin adsorbents ^8^	0.10	0.10	0.10	0.10	0.10
β-glucanase and β-xylanase ^9^	0.02	0.02	0.02	0.02	0.02
Calculated composition ^10^					
NE (kcal/kg)	2470	2470	2470	2470	2470
Lysine (%)	1.40	1.40	1.40	1.40	1.40
Methionine (%)	0.58	0.58	0.58	0.58	0.58
Methionine + cystine (%)	0.85	0.85	0.85	0.85	0.85
Threonine (%)	0.98	0.98	0.98	0.98	0.98
Tryptophan (%)	0.32	0.32	0.32	0.33	0.32
Valine (%)	0.97	0.97	0.97	0.97	0.97
Neutral Detergent Fiber (%)	10.44	10.43	10.44	10.56	10.56
Analyzed chemical composition ^11^, %					
Dry matter	89.28	89.48	89.73	89.81	89.57
Crude protein	17.21	17.82	17.48	17.14	17.84
Neutral detergent fiber	11.17	11.44	11.66	12.85	12.34
Acid detergent fiber	3.80	3.77	4.12	4.95	4.23
Acid detergent lignin	0.70	0.70	0.74	0.85	0.78
Total dietary fiber	13.51	14.13	14.76	13.28	14.92
Insoluble fiber	12.27	12.92	13.26	12.14	12.91
Soluble fiber	1.24	1.20	1.50	1.13	2.00

^1^ Dietary treatment: C−: negative control diet; C+: positive control diet; PRO: diet with probiotic; PROC: diet with probiotic plus 5% carob meal; PROP: diet with probiotic plus 5% citrus pulp. ^2^ HP-300 (Hamlet Protein, Horsens, Denmark). ^3^ Palbio 50 RD (Bioibérica SA, Barcelona, Spain). ^4^ INMUPROT 70, ingredient based on a blend of potato protein, egg meal, and soy-protein concentrate (INGASO FARM SL, Álava, Spain). ^5^ Miya-Gold^®^ S, microbiological feed additive (4b1830) containing *Clostridium butyricum* (*Clostridium butyricum Miyairi* 588 (FERM BP-2789), MIYA-GOLD^®^, Huvepharma^®^, Antwerp, Belgium); providing per kg diet: 2.5 × 10^8^ CFU. ^6^ ZINCOTRAX (Andrés Pintaluba SA, Tarragona, Spain); providing per kg of diet: 2500 mg Zn. ^7^ The premix supplied per kilogram of diet: vitamin A, 12,000 IU; vitamin D_3_, 2000 IU; vitamin E, 50 IU; vitamin K_3_, 2 mg; vitamin B_1_, 1.5 mg; vitamin B_2_, 4 mg; vitamin B_6_, 3 mg; vitamin B_12_, 0.035 µg; nicotinic acid, 26 mg; D-pantothenic acid, 15 mg; folic acid, 1 mg; choline, 200 mg; biotin, 0.18 mg; Fe, 125 mg as iron carbonate; Cu, 10 mg as copper sulfate pentahydrate; Zn, 120 mg as zinc oxide; Mn, 45 mg as manganese (II) oxide; I, 0.8 mg as calcium iodate; selenium, 0.3 mg as sodium selenite; 750 FYT as 6-Phytase EC 3.1.3.26. ^8^ TOXY-NIL^®^ Plus Dry (Nutriad, Turnhout, Belgium). ^9^ Axtra^®^ XB 201 TPT (Danisco Animal Nutrition, Madrid, Spain). ^10^ According to the Fundación Española para el Desarrollo de la Nutrición Animal [19]. ^11^ Each dietary treatment was analyzed in duplicate.

**Table 2:** Body weight, average daily gain (ADG), apparent ileal and fecal digestibility of dry matter and crude protein, and digesta pH values of piglets fed experimental diets.

	Diets ^1^						
Item	C−	C+	PRO	PROC	PROP	SEM ^2^	*p*-Value
Body weight (kg)							
0 d	4.96	4.91	5.15	5.13	5.08	0.150	0.979
14 d	7.29	7.14	7.78	7.43	7.74	0.211	0.842
26 d	11.23	10.48	11.93	10.99	11.97	0.368	0.669
ADG (kg/d)							
0–14 d	0.167	0.161	0.187	0.164	0.189	0.009	0.726
14–26 d	0.328	0.278	0.345	0.296	0.352	0.016	0.569
0–26 d	0.243	0.216	0.256	0.225	0.265	0.011	0.642
Apparent digestibility							
Ileal							
DM ^3^ (%)	65.27	66.45	67.09	67.32	66.51	1.734	0.991
CP ^4^ (%)	67.37	69.81	71.46	68.24	70.39	1.731	0.916
Fecal							
DM (%)	78.93 ^b^	83.80 ^a^	81.38 ^ab^	82.49 ^ab^	82.51 ^ab^	0.425	0.013
CP (%)	79.04	83.04	80.01	81.87	82.33	0.731	0.398
Digesta pH values							
Stomach	3.69	3.34	2.63	3.06	3.18	0.224	0.760
Duodenum	5.16	5.69	5.97	5.89	6.18	0.258	0.749
Jejunum	6.20	6.53	6.36	6.80	6.44	0.070	0.114
Ileum	6.80	7.01	7.05	7.24	6.72	0.088	0.376
Cecum	5.65	6.20	5.83	5.85	5.71	0.063	0.088
Colon	5.83	6.19	6.09	5.84	6.02	0.075	0.518

^1^ Dietary treatment—C−: negative control diet; C+: positive control diet; PRO: diet with probiotic; PROC: diet with probiotic plus 5% carob meal; PROP: diet with probiotic plus 5% citrus pulp. ^2^ SEM—standard error of the mean: *n* = 6 replicates per treatment. ^3^ DM—dry matter. ^4^ CP—crude protein. ^ab^ Means with different superscripts in the same row are different (*p* < 0.05).

**Table 3:** Microbiology profile of feces from piglets fed the experimental diets at 26 days post-weaning.

	Diets ^1^						
Item	C−	C+	PRO	PROC	PROP	SEM ^2^	*p*-Value
Bacterial population							
(log_10_ CFU/g feces)							
*Enterobacteriaceae*	5.89	6.18	5.63	6.11	5.50	0.287	0.929
Coliform bacteria	5.80	6.14	5.48	6.05	5.31	0.282	0.859
Lactic acid bacteria	7.30	6.95	7.94	7.46	7.48	0.145	0.135

^1^ Dietary treatment—C−: negative control diet; C+: positive control diet; PRO: diet with probiotic; PROC: diet with probiotic plus 5% carob meal; PROP: diet with probiotic plus 5% citrus pulp. ^2^ SEM—standard error of the mean: *n* = 6 replicates per treatment.

**Table 4:** Concentration of volatile fatty acids (VFAs) and fermentation profile in cecum and feces from piglets fed the experimental diets.

	Diets ^1^						
Item	C−	C+	PRO	PROC	PROP	SEM ^2^	*p*-Value
Cecum							
Total VFAs (mmol/kg)	93.44 ^a^	55.54 ^b^	76.38 ^ab^	78.72 ^ab^	77.96 ^ab^	3.868	0.066
Acetic acid (molar %)	57.35	60.97	55.28	59.05	57.89	1.304	0.740
Propionic acid (molar %)	24.89	20.21	24.67	22.68	22.94	0.672	0.213
Butyric acid (molar %)	13.41	12.45	14.10	13.10	14.33	0.854	0.961
Isobutyric acid (molar %)	0.30	0.97	0.43	0.68	0.33	0.090	0.192
Isovaleric acid (molar %)	0.28	1.16	0.42	0.70	0.31	0.111	0.115
Valeric acid (molar %)	3.74	4.21	5.07	3.76	4.17	0.425	0.856
Feces							
Total VFAs (mmol/kg)	105.02 ^ab^	66.76 ^b^	93.18 ^ab^	121.42 ^a^	114.90 ^ab^	5.630	0.049
Acetic acid (molar %)	71.41 ^ab^	74.41 ^a^	67.25 ^ab^	64.26 ^b^	68.43 ^ab^	0.934	0.031
Propionic acid (molar %)	15.65	16.43	16.62	17.83	16.88	0.494	0.705
Butyric acid (molar %)	5.36 ^ab^	3.16 ^b^	8.67 ^a^	8.95 ^a^	7.96 ^a^	0.495	0.009
Isobutyric acid (molar %)	1.82	1.79	2.04	1.97	1.69	0.192	0.979
Isovaleric acid (molar %)	2.40	2.32	2.60	2.54	2.09	0.284	0.982
Valeric acid (molar %)	3.35	1.87	2.81	4.41	2.92	0.302	0.152

^1^ Dietary treatment—C−: negative control diet; C+: positive control diet; PRO: diet with probiotic; PROC: diet with probiotic plus 5% carob meal; PROP: diet with probiotic plus 5% citrus pulp. ^2^ SEM—standard error of the mean: *n* = 6 replicates per treatment. ^ab^ Means with different superscripts in the same row are different (*p* < 0.05).

**Table 5:** Histomorphometrical and immunohistochemical intestinal parameters from piglets fed the experimental diets.

	Diets ^1^						
Item	C−	C+	PRO	PROC	PROP	SEM ^2^	*p*-Value
Jejunum							
Villus height (µm)	508.72	422.90	551.92	481.22	555.67	16.656	0.151
Crypt depth (µm)	237.42	288.40	266.90	267.45	271.12	6.148	0.202
Villus/crypt ratio	2.23	1.50	2.08	1.82	2.09	0.083	0.103
Goblet cells ^3^	0.50	0.96	0.40	0.30	0.40	0.094	0.304
Lymphocytes ^3^	18.70	19.93	18.35	15.92	15.55	0.825	0.437
IgA cells ^3^	10.25	11.10	14.225	14.12	9.47	0.839	0.260
Ileum							
Villus height (µm)	411.70	426.83	485.40	409.42	427.70	15.756	0.534
Crypt depth (µm)	219.15	249.50	256.60	228.92	256.70	7.866	0.438
Villus/crypt ratio	1.92	1.72	1.90	1.85	1.73	0.066	0.824
Goblet cells	1.05	1.13	0.80	0.92	0.95	0.138	0.955
Lymphocytes	15.70	14.23	16.40	15.37	14.05	0.612	0.713
IgA cells	11.47	6.53	8.87	10.47	6.07	1.008	0.388

^1^ Dietary treatment—C−: negative control diet; C+: positive control diet; PRO: diet with probiotic; PROC: diet with probiotic plus 5% carob meal; PROP: diet with probiotic plus 5% citrus pulp. ^2^ SEM—standard error of the mean: *n* = 6 replicates per treatment. ^3^ Number of cells/25,000 µm^2^.

**Table 6:** General biochemical parameters, oxidative balance indicators and cytokines in serum of piglets feed with experimental diets at 25 days post-weaning.

	Diets ^1^						
Item	C−	C+	PRO	PROC	PROP	SEM ^2^	*p*-Value
Biochemical parameters							
Glucose (mg/dL)	83.60	81.40	91.22	85.35	92.70	2.932	0.759
Urea (mg/dL)	27.25	25.70	28.53	26.98	28.50	1.029	0.848
Cholesterol (mg/dL)	89.07	74.97	80.25	76.25	92.57	4.258	0.626
Triglycerides (mg/dL)	45.13	31.73	31.74	41.34	33.99	2.567	0.417
Total Bilirubin (mg/dL)	0.230	0.197	0.170	0.165	0.195	0.014	0.625
Total Proteins (g/dL)	4.81	4.65	4.80	5.20	4.98	0.110	0.620
Albumin (g/dL)	2.48	2.16	2.37	2.57	2.65	0.080	0.348
Albumin/Globulin ratio	1.07	0.87	0.97	1.00	1.14	0.042	0.361
(AST) (IU/L) ^3^	94.44	65.75	68.82	65.44	75.92	5.593	0.525
(ALT) (IU/L) ^4^	89.87 ^ab^	115.42 ^a^	86.40 ^ab^	71.30 ^b^	84.05 ^ab^	4.393	0.089
Oxidative balance indicators							
TOS (µmol/L) ^5^	13.94	12.05	8.57	10.35	11.50	1.286	0.762
TAC (mmol/L) ^6^	0.824	0.776	0.768	0.746	0.777	0.010	0.227
Cytokines							
IL-1β (ng/mL)	0.064	0.144	0.061	0.091	0.096	0.020	0.803
IL- 6 (ng/mL)	0.018	0.088	0.018	0.031	0.034	0.014	0.628
IL- 8 (ng/mL)	3.346 ^a^	2.992 ^a^	3.043 ^a^	1.145 ^b^	2.219 ^ab^	0.272	0.036
IL-10 (ng/mL)	0.389	1.958	0.252	0.384	1.022	0.358	0.641
IL- 12 (ng/mL)	3.453	3.071	3.835	3.249	2.749	0.196	0.448
TNF-α (ng/mL)	0.080	0.078	0.078	0.086	0.088	0.002	0.627

^1^ Dietary treatment—C−: negative control diet; C+: positive control diet; PRO: diet with probiotic; PROC: diet with probiotic plus 5% carob meal; PROP: diet with probiotic plus 5% citrus pulp. ^2^ SEM—standard error of the mean: *n* = 6 replicates per treatment. ^3^ AST—aspartate aminotransferase. ^4^ ALT—alanine aminotransferase. ^5^ TOS—total oxidant status. ^6^ TAC—total antioxidant capacity. ^ab^ Means with different superscripts in the same row are different (*p* < 0.05).

## Data Availability

Data sharing is not applicable to this article.
